# Comparison of machine-learning methodologies for accurate diagnosis of sepsis using microarray gene expression data

**DOI:** 10.1371/journal.pone.0251800

**Published:** 2021-05-17

**Authors:** Dominik Schaack, Markus A. Weigand, Florian Uhle

**Affiliations:** Department of Anesthesiology, Heidelberg University Hospital, Heidelberg, Germany; Northeast Normal University, CHINA

## Abstract

We investigate the feasibility of molecular-level sample classification of sepsis using microarray gene expression data merged by in silico meta-analysis. Publicly available data series were extracted from NCBI Gene Expression Omnibus and EMBL-EBI ArrayExpress to create a comprehensive meta-analysis microarray expression set (meta-expression set). Measurements had to be obtained via microarray-technique from whole blood samples of adult or pediatric patients with sepsis diagnosed based on international consensus definition immediately after admission to the intensive care unit. We aggregate trauma patients, systemic inflammatory response syndrome (SIRS) patients, and healthy controls in a non-septic entity. Differential expression (DE) analysis is compared with machine-learning-based solutions like decision tree (DT), random forest (RF), support vector machine (SVM), and deep-learning neural networks (DNNs). We evaluated classifier training and discrimination performance in 100 independent iterations. To test diagnostic resilience, we gradually degraded expression data in multiple levels. Clustering of expression values based on DE genes results in partial identification of sepsis samples. In contrast, RF, SVM, and DNN provide excellent diagnostic performance measured in terms of accuracy and area under the curve (>0.96 and >0.99, respectively). We prove DNNs as the most resilient methodology, virtually unaffected by targeted removal of DE genes. By surpassing most other published solutions, the presented approach substantially augments current diagnostic capability in intensive care medicine.

## Introduction

Understanding of the syndrome sepsis as a complicated disturbance of core immune functions has advanced in the last years, resulting in improved outcomes for affected patients. According to the latest Sepsis-3 definition, the syndrome is described as a life-threatening condition arising from the body’s response to an infection injuring its tissues and organs. Since sepsis is still related to high death rates and severe long-term effects [1], precise and reliable diagnosis and accurately timed treatment is fundamental to successfully prevent patients from poor outcomes [2, 3].

To reach a final decision on diagnosing complex diseases like sepsis, it is essential for physicians to precisely consider a vast collection of clinical information for each case. The utility of machine-learning-based methods to support physicians in the identification of sepsis patients has been evaluated and was demonstrated to show higher performance to predict sepsis 3–4 hours before its onset compared to existing scoring systems like modified early warning score (MEWS), sequential organ failure assessment (SOFA), and the simplified quickSOFA (qSOFA) [4]. Besides, the increased availability of clinical routine and intensive care unit (ICU) data lead most previous studies [5] to focus their research on data-intensive resources like vital signs or electronic health record (EHR) data.

Concurrently precision medicine emerges as a novel comprehensive approach to access information offered by molecular diagnostics. With sepsis, gene expression profiles could help identify and detect transcriptomic sub-phenotypes (endotypes) to create individually tailored therapy strategies, e.g., severe immune suppression [6]. By offering standardized and usable methodologies for molecular diagnostics, biological markers have been developed as indicators for complex diseases, effectively tracing gene expression activity for a limited set of informative genes. In recent developments, the number of observed genes is increased to enable the assembly of a comprehensive molecular signature to detect disease-related processes. In contrast to widely used RT-PCR measurement of a small set of selected biomarkers, both microarray and ribonucleic acid sequencing (RNA-seq) technologies comprise, through the high number of available genes, an enormous informational content and thus could provide several new informative features for diagnosis. With the most comprehensive set of genes available and discrete gene expression measurement, RNA-seq certainly is a promising technology. While being comparable to RNA-seq concerning high-dimensional measurements of gene expression, microarrays are, in contrast, publicly available to date for numerous datasets. Therefore, we developed an automated solution for microarray gene expression analysis to make their features accessible for the sake of medical diagnosis.

Conventional bioinformatic analyses on high-dimensional gene expression datasets aim to delineate differentially expressed genes by comparing predefined groups. By applying arbitrary thresholds, the most promising candidate genes are condensed to informative gene signatures. While those results yield important pathophysiological insights into the biological processes, the conversion of the result to a forward-oriented diagnostic classifier demands extensive steps: defining a minimal gene set of maximal informational content, incorporating individual weights for each gene and the interaction, iterative validation, and optimization of the tool. Overall, this approach possesses inherent pitfalls, not least, the inevitable loss of information and the risk of selecting “wrong” genes.

Therefore, we asked if conventional machine-learning algorithms might be a superior straight-forward approach to establish a robust diagnostic framework, able to handle high-dimensional gene expression data without the requirement of preprocessing or selection and thus circumventing many drawbacks of conventional bioinformatics analysis.

This publication implements automated sepsis prediction and sample classification on a molecular level for diagnosis using microarray-derived gene expression data. To assess feasibility and performance, we performed differential expression analysis and applied and compared different well-established machine-learning-based methodologies for the aim of accurate and reliable diagnosis.

## Material and methods

### Dataset retrieval

Machine learning has been demonstrated as most effective when used for analysis of high-dimensional datasets [7]. To create a large-scale microarray collection of sepsis-related samples, we gathered 214 candidate data series from NCBI Gene Expression Omnibus (GEO) and 157 from EMBL-EBI ArrayExpress, respectively. We selected candidate data series according to five criteria: Measurements had to be obtained (i) via microarray-technique from (ii) whole blood samples of (iii) adult or pediatric patients with (iv) sepsis that has been diagnosed based on international consensus definition (v) immediately after admission to ICU. After careful review of all candidates, a consistent collection of 22 data series was created [8–27]. Overall, our data series collection comprises 1,354 sepsis samples, 86 patients with SIRS, and 346 healthy individuals available for classification (S1 File, upper section).

To challenge our machine learning approach, we intentionally added trauma patients’ samples with no infection signs to the data collection. The differential diagnosis of sepsis and trauma from gene expression data is a challenging issue attributed to the indifferent immunological phenotype, based on the comparable underlying transcriptional response [28]. This study thus included all available microarray-based blood samples of trauma-related GEO data series GSE36809 [29]. After careful examination of the associated metadata, we could identify 185 individuals in total with severe blunt trauma besides the 35 healthy controls in the data series. However, the publication itself describes just 167 trauma patients. As a second source of trauma samples, data series GSE37069 [30] was evaluated. According to the original study, GSE37069 should comprise 244 burn trauma individuals and 35 control samples. We found microarrays from 248 trauma patients and 37 controls to be retrievable from GEO, while controls fully overlapped with the sample identifiers from GSE36809 and thus were excluded from further analysis. Besides, data series GSE19743 [31] and GSE77791 [32] were identified to hold further 87 trauma patients and 76 associated healthy control samples. With four trauma-related data series appended to the data collection, the final dataset comprises 26 originating data series. Overall, we added 520 patients diagnosed with trauma and 111 healthy controls to the existing samples (S1 File, lower section).

Concerning 1,354 septic samples, a limited number of 520 samples with trauma are available. In general, if one class is represented by many examples, while only a few represent the other, class imbalance emerges and can lead to significant performance issues for classification [33]. To avoid the drawbacks of matching the positive class of 1,354 sepsis patients to an over 60% smaller set of trauma-related samples, we created a composite negative classification entity originating from the available 457 associated healthy controls and all 86 and 520 patients diagnosed with SIRS and trauma, respectively. With the goal of binary classification, consolidation of the entire non-septic samples to one entity harmonizes sample numbers of both classes (1,354 sepsis patients vs. 1,063 non-septic samples) (S1 File). Collectively about 56% of the collected samples originate from sepsis patients, while the non-septic entity provides approximately 44% of all samples leading to an almost equal distribution for both classes.

### Data preparation

All selected data series were merged into a meta-expression set to ensure compatibility between microarray platforms. The merging process is described in [34]. We performed data preparation and analysis in R/Bioconductor [35] environment. If the expression of a single gene is covered by more than one microarray probe, we averaged the calculated probe expression values to maximize information content. Since merged data was limited to the least common denominator of genes between included microarray platforms, all expression values adducted for analysis are based on real data points. We did not substitute missing data with imputation techniques. The fully merged meta-expression set contains 5,932 gene expression values of 2,417 samples.

It is essential to perform inter-study normalization of gene expression data between all 26 included data series as a prerequisite of differential expression analysis. Since GEO data series GSE25504 comprises samples originating from four different microarray platforms, we had to extend batch effect adjustment to 29 cohorts. Subsequently, ComBat [36] method was applied considering subjects’ relationships to both originating data series and microarray platforms.

For machine learning algorithms, in contrast to an elaborate batch effect adjustment routine, the preprocessing for the entire meta-expression dataset limited to a two-step procedure. First, gene-wise subtraction of the mean value from all individual expression values (centering) was applied. Subsequently, centered expression values were divided per gene by their standard deviation (scaling).

### Differential expression analysis

Differential expression analysis between sepsis and non-sepsis patients was obtained by limma [37]. The software was chosen based on its ability to support complex experimental settings with a simultaneous comparison of many microarray targets. The package’s strong and versatile capabilities for microarray differential expression assessment are based on linear models. The lowest absolute LogFC value between conditions required for considering genes to be differentially expressed was 1.0 with simultaneous consideration of statistical significance (adjusted p-values below 0.05). We carried out the Benjamini-Hochberg procedure to perform correction for multiple testing.

### Implementation of machine-learning methodologies

For implementing automated sepsis diagnosis, we chose four machine-learning methods: Decision tree (DT) and random forest (RF), both generating tree-based models for class determination, and the more complex support vector machine (SVM) and deep-learning neural network (DNN) classifiers to consider recent developments in artificial intelligence (AI). Data analysis and processing with DT, RF, and SVM were based on the CRAN R packages “tree” (cran.r-project.org/web/packages/tree), “randomForest” (cran.r-project.org/web/packages/randomForest), and “e1071” (cran.r-project.org/web/packages/e1071) using the recommended parameters specific to the respective methodology. As the framework for DNN, we chose Google TensorFlow [38], which we accessed through the “keras” package [39].

We performed all training, validation, and testing steps by TensorFlow’s graphics processing unit (GPU) implementation to shorten the run time for calculations. All other machine-learning procedures are computationally less extensive and could be entirely executed on the workstation’s central processing unit (CPU).

### Architecture of the deep-learning-based classifier

We adapted the neural network’s architecture to enforce generalized classification for the samples provided and avoid over-fitting the classifier to idiosyncrasies of the underlying meta-expression set. Therefore, additional dropout layers [40] and an L2 kernel regularizer [41] were included. Both changes affect classifier training performance: Dropout layers randomly prevent the majority of neurons from processing and transferring information to the consecutive layers, while L2 kernel regularization penalizes the emergence of high weight values. The input and hidden layers use a rectified linear unit (ReLU) [42] activation function, while the output layer uses Sigmoid Function, respectively. Penalty (error) is described by categorical cross-entropy function. We carried out stochastic gradient-based optimization using the AdaMax optimizer [43].

The best performing architecture (reported as the accuracy of classification) given the data provided is a five-layer neural network (multi-layer perceptron) with one hidden layer at its center and two adjacent dropout layers between the essential input and output layers.

### Workflow for sepsis prediction

For predicting sepsis syndrome with established machine-learning-based methodologies, we gradually execute the following five-step procedure: (1) The complete meta-expression set is split into two parts to provide subsets for classifier training and testing. Regarding training data, approximately 85% of the available samples are evenly and randomly drawn from the included septic patients, and the composite group of non-septic samples. For classifier testing, 15% of the entire meta expression samples are assembled accordingly. (2) The classifiers are constructed in a training process based on information extracted from the combination of supplied samples, and their respective class labels. (3) Trained classifiers are utilized to predict the class affiliation of all samples taken from the previously separated testing subset (4) Results are compared to the correct class labels, and predictive metrics are reported. (5) Finally, constructed classifiers and randomized expression data subsets are discarded to enable unbiased replication of the procedure. For each method, we performed 100 independent iterations.

For DNNs, we conduct an adapted and slightly more complex workflow: (1) The meta-expression set is split into three parts arranging subsets for classifier training, validation, and testing. 80% of all samples are prepared as a training subset following the description above. Subsets for validation and testing receive the remaining 5% and respectively 15% of all available samples. (2) The neural network is trained in multiple epochs using the corresponding independent, randomly composed subsets. While training, initially randomized values for the connection-assigned weights are gradually adapted to minimize error and maximize prediction accuracy by comparing results to the existing true class labels. Validation metrics calculated at the end of each epoch were exclusively used to guide initial model prototyping. During training for final classifier evaluation, no callback functions for automated assessment of validation data were employed. (3) Prediction of the previously separated testing subset is facilitated using the trained neural network. We again performed 100 independent iterations.

Subsequent performance reporting and re-initialization steps are conducted according to the descriptions mentioned above.

### Stratified ShuffleSplit cross-validation and evaluation of classification performance

Since the meta-expression set is still slightly imbalanced regarding the number of samples in each class, by evenly drawing septic and non-septic samples, we realized a stratified sampling strategy to ensure the required equal representation of both classes for training, validation (for DNNs), and testing. With the iterative repetition of the entire approach, Stratified ShuffleSplit cross-validation is implemented to prove the reproducibility of classification results.

After completion of training and prediction steps during each iteration, predictive metrics (area under the curve (AUC) and probability of correct classification (PCC)) are calculated based on the respective machine-learning classifier results, and receiver operating characteristic (ROC) plots are generated using R package “PresenceAbsence” [44]. The difference between AUC and PCC means was compared by unpaired Student’s t-tests using R base functions for all machine-learning-based predictions.

### Mechanisms of artificial data degradation

**Fig 1:**
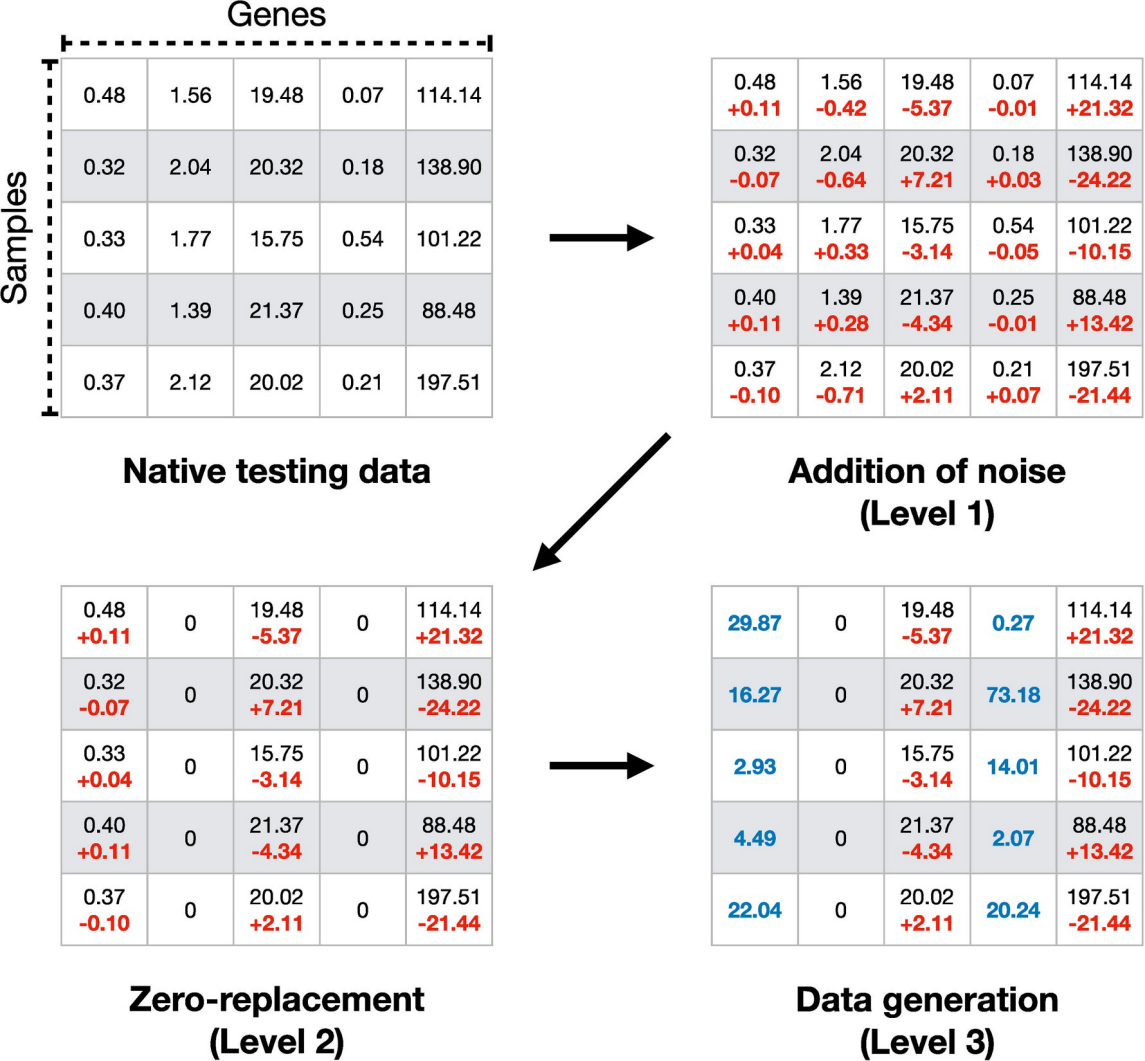
Mechanisms of data degradation. Data manipulation is shown for a random sample dataset. Native testing data are randomly degraded in three cumulative levels. First, noise is added to all expression values to generate minor fluctuations. At the second level, gene expression information is removed by zero-replacement. In the last step, simulated but plausible data replaces both real expression values and zero-replaced genes.

In general, training (and validation in DNNs) is facilitated with subsets of samples holding unaltered expression data. To test the resilience and flexibility of the four machine-learning-based classifiers, we introduced virtual obstacles. Therefore, testing data was altered to resemble real-world issues frequently emerging in gene expression data, e.g., arising from technical and methodological bias. We modified expression values in a sequence of three consecutive levels of signal degradation (Fig 1): (1) Noise is added to all 5,932 gene expression values of every test sample by using the R base function “jitter” with the factor parameter set to 10,000. (2) The noise-added test data signal is further dispersed by a randomized replacement of 75% of the available gene expression values by zero, effectively retaining a limited subset of 1,483 genes for classification. (3) 50% of all genes (2,966) are randomly selected to be replaced with simulated expression data. Data is generated gene-wise by R stats function “rnorm” based on the median of expression values over all samples in the testing subset and a standard deviation parameter of 20 to ensure plausibility. The number of genes replaced during steps 2 and 3 is chosen arbitrarily with the rationale to clearly illustrate the effects of gene expression information sparsity and simulated data misguidance on classifier performance.

### Software reporting

Software components used for preprocessing of merged meta-datasets, batch effect adjustment, differential expression analysis, classifier training, analysis, and reporting are described in S2 File.

### Statement of ethical approval

Within this study, we reanalyzed publicly accessible datasets from repositories. Responsibility for the necessary provision of ethics approval and participation consent is signed over to the original data authors.

## Results

### Conventional gene expression analysis

**Fig 2:**
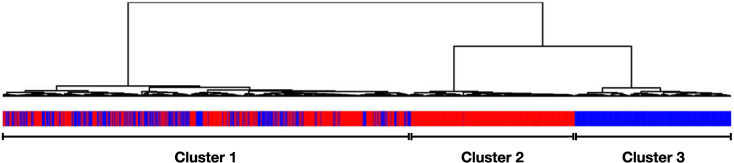
Dendrogram of clustering results based on differentially expressed genes. Custom annotation illustrates the actual sample classification. Hierarchical clustering based on derived expression data identifies three main sub-groups (from left to right), with the first cluster containing both samples with sepsis patients (red) and non-sepsis samples (blue), a second cluster almost entirely comprising sepsis (red), and a third cluster exclusive to non-sepsis samples (blue).

We applied differential expression analysis based on batch-effect adjusted data in a first experiment using the complete meta-expression set. The contrast was defined to compare differences between the first class of samples diagnosed with sepsis and the entity of non-septic patients as second class. In this comparison, 2,361 genes were reported to be differentially expressed (Log transformed fold change (LogFC) ±1; adjusted p-values <0.05). Agglomerative hierarchical clustering of all samples (Euclidean distance, Ward 2 criterion) based on these differentially expressed genes identifies three main subgroups of samples (Fig 2). The most apparent result is the aggregation of non-septic samples as an exclusive subgroup in the resulting sample dendrogram. A second, almost equally sized subgroup comprises almost solely sepsis patients. The third subgroup contains a larger number of samples and represents samples of both classes. We can conclude that with clustering of batch effect adjusted gene expression data, it is possible to distinguish parts of the non-septic samples from the remaining collective. However, the method’s inability to clearly distinguish between sepsis patients and the entity of non-sepsis samples is explicitly demonstrated by the existence of a vastly unorganized subgroup comprising samples of both classes. Based on these results, we refrained from conventional differential analysis of microarray gene expression data and evaluated the performance of different machine-learning-based methods for the aim of reliable diagnosis.

### Diagnostic classification by machine learning

**Fig 3:**
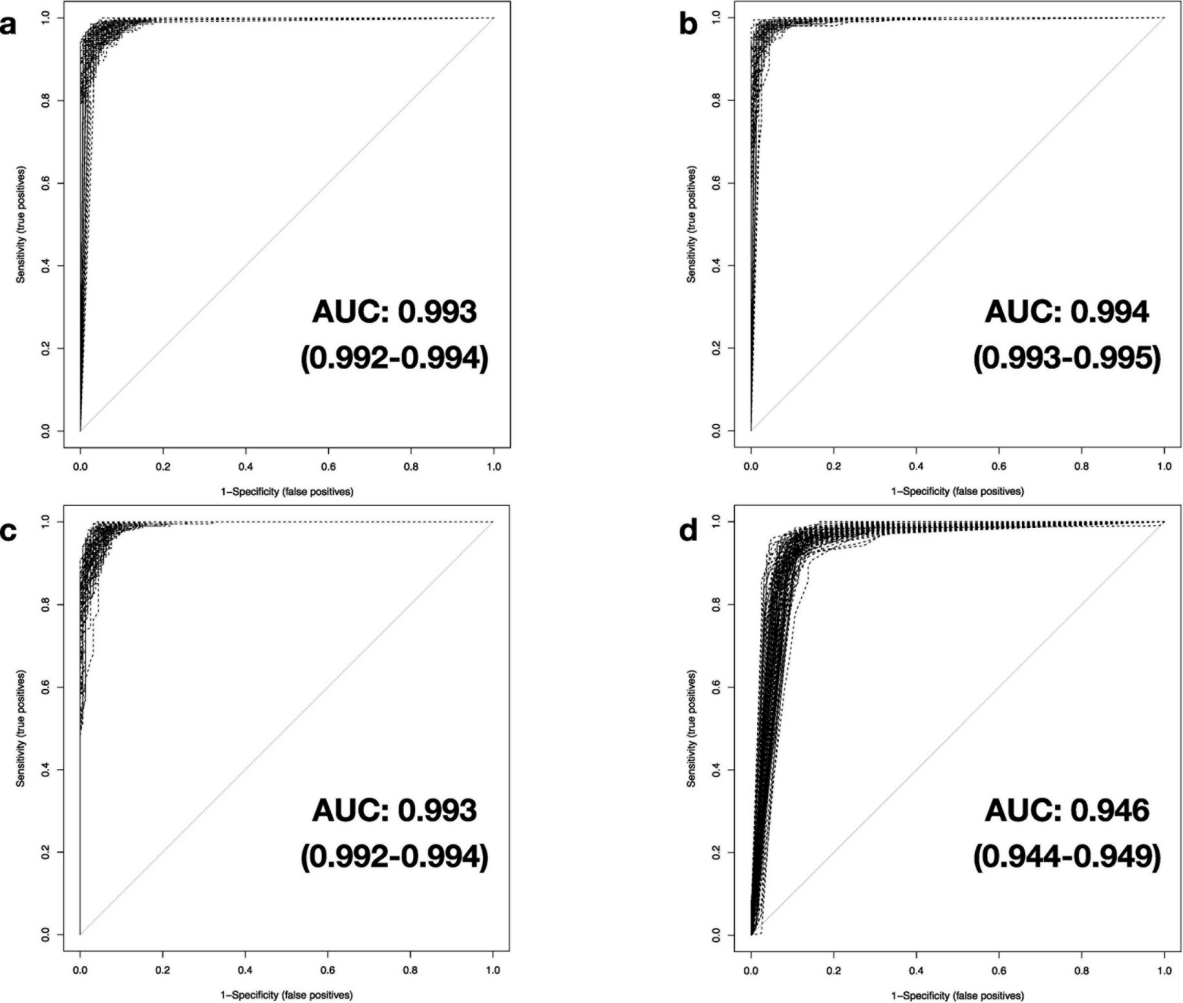
Classification performance of investigated machine-learning-based methods. Results of the AUROC analyses for performance measurement of the trained classifiers in 100 iterations based on native testing data (5,932 genes) for a) DNN, b) SVM, c) RF, and d) DT. Results of mean AUC and confidence intervals (CI, in parentheses) at 95% confidence level are shown.

Four different methodologies were tested for their performance to generate a precise binary classifier between sepsis patients and non-septic samples, based on labeled, preprocessed expression data originating from the meta-expression set. We subsequently evaluated classifier performance on unknown samples by comparing the assignment results to the true class. Fig 3 shows the resulting multiplex receiver operating characteristic (ROC) plot for 100 independent iterations. All algorithms exhibit very high performance assessed by their corresponding AUC and PCC (Tables 1 and 2). With unaltered test data, RF, SVM, and DNNs deliver comparable performance with mean values of AUC and PCC of over 0.99 and 0.96, respectively. The lesser complex DT methodology shows slightly weaker results over all iterations, still providing very high diagnostic performance (mean AUC: 0.946 (0.944–0.949), mean PCC: 0.924 (0.922–0.927)).

**Table 1:** Detailed comparison of diagnostic performance (by AUC) for investigated machine-learning classifiers.

Full dataset					Reduced dataset				
Procedure			p-value		Procedure			p-value	
Native	**Mean AUC**	**95% CI**	**vs. native**	**vs. DNN**	Native	**Mean AUC**	**95% CI**	**vs. native**	**vs. DNN**
DNN	0.993	0.992–0.994			DNN	0.994	0.993–0.994		
SVM	0.994	0.993–0.995		3.1E-02	SVM	0.993	0.992–0.993		3.8E-02
RF	0.993	0.992–0.994		9.6E-01	RF	0.993	0.992–0.993		1.0E-02
DT	0.946	0.944–0.949		2.7E-61	DT	0.938	0.935–0.941		1.3E-60
Level 1 deg.	**Mean AUC**	**95% CI**			Level 1 deg.	**Mean AUC**	**95% CI**		
DNN	0.990	0.989–0.991	2.6E-11		DNN	0.987	0.987–0.988	4.5E-23	
SVM	0.946	0.944–0.948	3.3E-70	4.3E-67	SVM	0.919	0.916–0.922	9.9E-80	3.6E-78
RF	0.888	0.883–0.892	1.5E-72	1.9E-71	RF	0.853	0.849–0.857	6.2E-87	2.1E-86
DT	0.634	0.627–0.641	5.5E-107	3.8E-100	DT	0.596	0.59–0.602	1.5E-129	9.3E-114
Level 2 deg.	**Mean AUC**	**95% CI**			Level 2 deg.	**Mean AUC**	**95% CI**		
DNN	0.964	0.961–0.967	4.0E-37		DNN	0.945	0.942–0.948	3.4E-52	
SVM	0.776	0.764–0.789	7.2E-57	6.1E-53	SVM	0.737	0.726–0.748	1.1E-67	6.7E-63
RF	0.886	0.881–0.89	4.2E-69	1.1E-64	RF	0.818	0.813–0.824	1.8E-82	1.7E-83
DT	0.570	0.559–0.582	5.7E-87	3.1E-90	DT	0.544	0.535–0.553	1.3E-104	2.9E-109
Level 3 deg.	**Mean AUC**	**95% CI**			Level 3 deg.	**Mean AUC**	**95% CI**		
DNN	0.818	0.809–0.826	2.6E-64		DNN	0.768	0.761–0.775	1.5E-80	
SVM	0.615	0.604–0.626	5.9E-86	1.5E-71	SVM	0.601	0.592–0.609	4.8E-96	5.1E-71
RF	0.773	0.764–0.781	3.6E-74	2.4E-12	RF	0.700	0.692–0.708	3.8E-88	3.0E-26
DT	0.539	0.53–0.548	8.5E-105	8.0E-104	DT	0.525	0.518–0.531	3.3E-134	2.3E-111

**Table 2:** Comparison of diagnostic accuracy (by PCC) for investigated machine-learning classifiers.

Full dataset					Reduced dataset				
Procedure			p-value		Procedure			p-value	
Native	**Mean PCC**	**95% CI**	**vs. native**	**vs. DNN**	Native	**Mean PCC**	**95% CI**	**vs. native**	**vs. DNN**
DNN	0.962	0.96–0.964			DNN	0.964	0.962–0.966		
SVM	0.972	0.971–0.974		1.9E-13	SVM	0.969	0.968–0.971		1.2E-05
RF	0.965	0.963–0.966		6.3E-02	RF	0.963	0.962–0.965		8.8E-01
DT	0.924	0.922–0.927		1.8E-55	DT	0.922	0.92–0.924		2.9E-67
Level 1 deg.	**Mean PCC**	**95% CI**			Level 1 deg.	**Mean PCC**	**95% CI**		
DNN	0.953	0.951–0.955	7.7E-11		DNN	0.947	0.945–0.949	4.7E-19	
SVM	0.884	0.88–0.887	1.9E-85	3.1E-75	SVM	0.849	0.846–0.852	5.1E-104	1.9E-100
RF	0.808	0.803–0.813	9.7E-89	2.9E-86	RF	0.772	0.768–0.777	1.9E-110	2.8E-113
DT	0.642	0.636–0.648	4.6E-115	3.2E-111	DT	0.594	0.588–0.6	1.3E-130	4.2E-128
Level 2 deg.	**Mean PCC**	**95% CI**			Level 2 deg.	**Mean PCC**	**95% CI**		
DNN	0.906	0.901–0.91	8.1E-50		DNN	0.881	0.877–0.886	1.9E-69	
SVM	0.717	0.706–0.728	1.6E-69	3.1E-62	SVM	0.688	0.678–0.697	5.2E-79	7.6E-70
RF	0.810	0.805–0.815	2.0E-86	1.8E-68	RF	0.746	0.741–0.752	1.4E-101	7.9E-91
DT	0.601	0.591–0.61	1.0E-91	9.6E-99	DT	0.572	0.566–0.578	2.4E-128	2.4E-140
Level 3 deg.	**Mean PCC**	**95% CI**			Level 3 deg.	**Mean PCC**	**95% CI**		
DNN	0.756	0.749–0.763	2.1E-82		DNN	0.713	0.707–0.72	7.0E-101	
SVM	0.600	0.592–0.609	6.7E-97	6.1E-67	SVM	0.588	0.58–0.595	9.3E-110	1.7E-65
RF	0.709	0.701–0.716	2.4E-89	2.6E-16	RF	0.652	0.645–0.658	1.6E-106	2.3E-29
DT	0.567	0.557–0.576	5.1E-97	3.3E-76	DT	0.550	0.544–0.557	2.2E-124	4.7E-86

### Resilience testing

**Fig 4:**
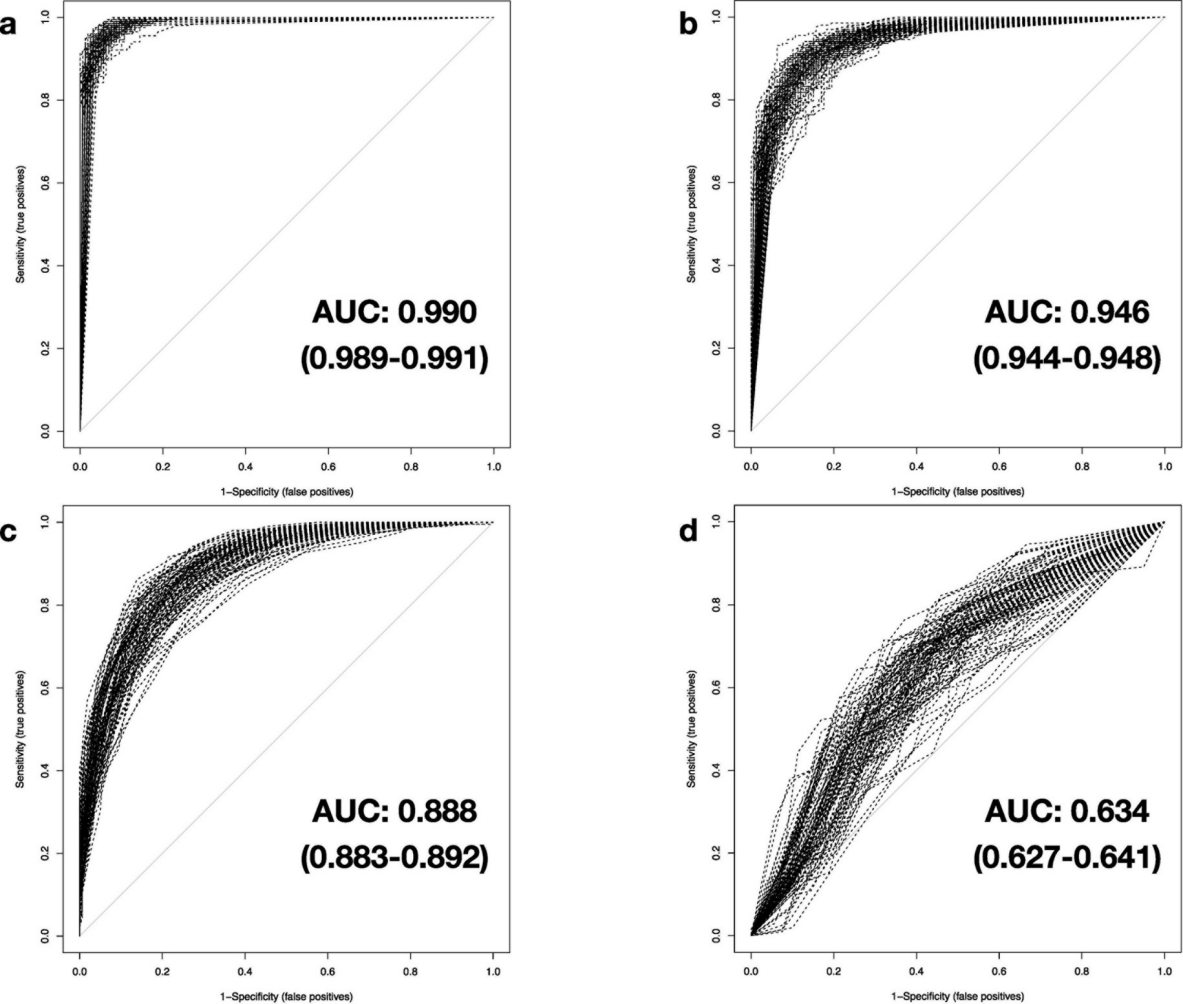
Comparison of classification performance with noise added to testing data. The AUROC analyses are based on testing data with level 1 data degradation applied on all 5,932 genes for a) DNN, b) SVM, c) RF, and d) DT.

**Fig 5:**
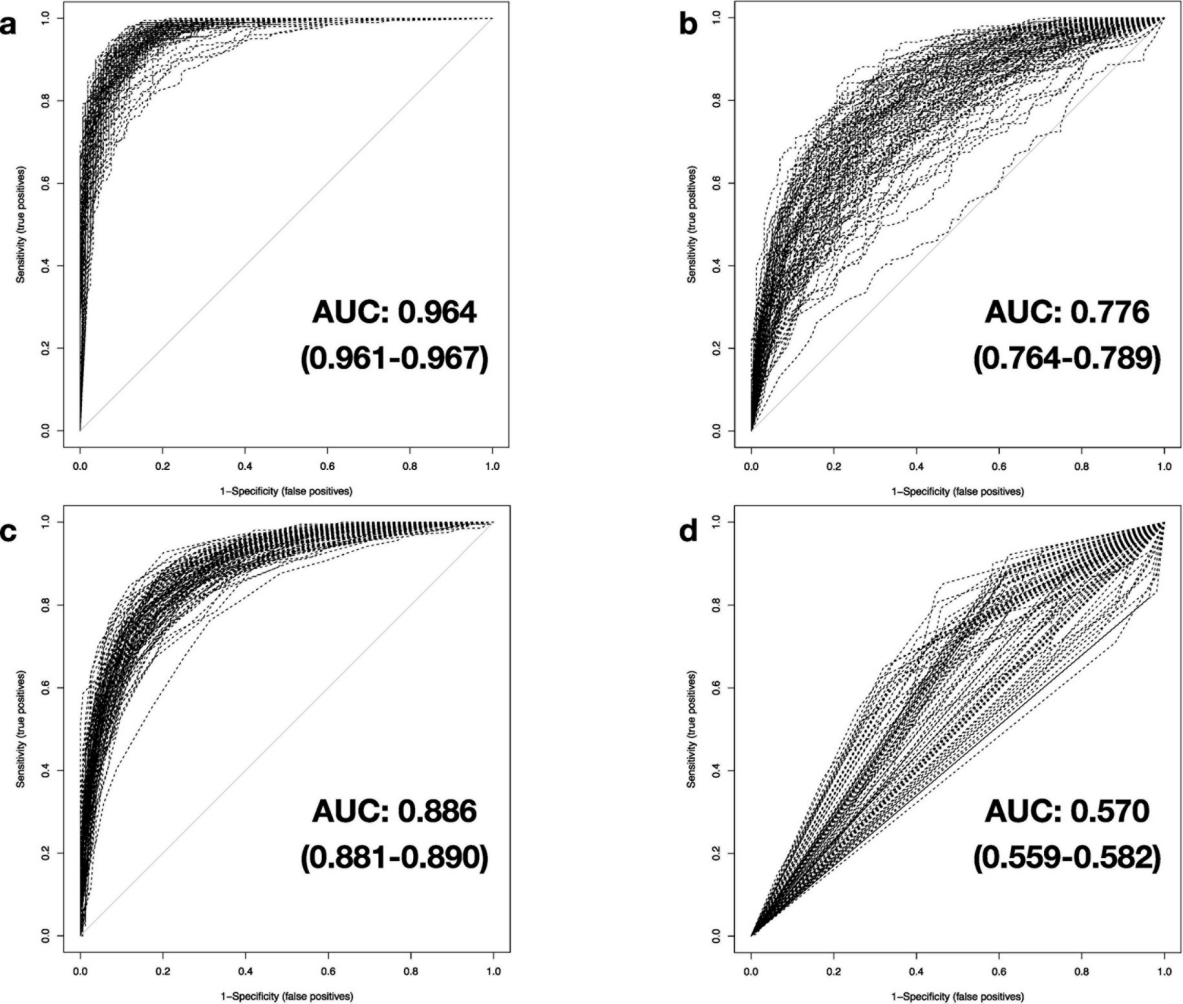
Comparison of classification performance with zero-replacement of 75% of testing data. The AUROC analysis results are derived from testing data with level 2 data degradation applied cumulatively to level 1, considering 1,483 genes for a) DNN, b) SVM, c) RF, and d) DT.

**Fig 6:**
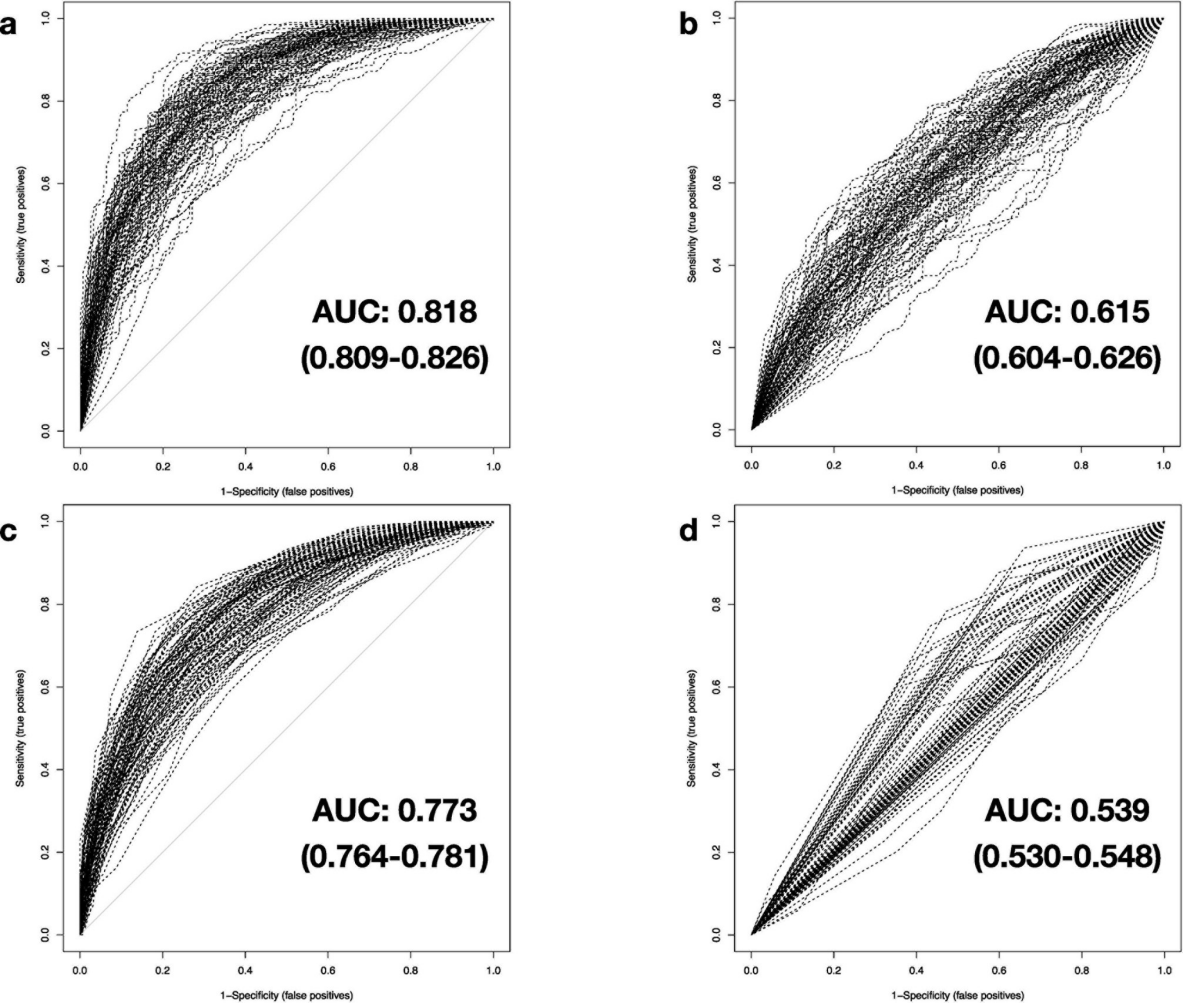
Comparison of classification performance with 50% simulated testing data. The results of the AUROC analysis are based on testing data with level 3 data degradation applied cumulatively to levels 1 and 2, examining simulated expression data of 2,966 genes in combination with real expression values of up to 1,483 genes for a) DNN, b) SVM, c) RF and d) DT.

To test the implementation’s resilience and reveal limitations between the machine-learning methods, we retried the last experiment under the same conditions but using virtual obstacles to simulate real-world bias. The testing samples’ expression data were incrementally degraded in three steps, while the training subset’s gene expression values were left unchanged.

(I) At the first level of test data modification, random noise is added to all original gene expression values, imitating the occurrence of a sample- or batch-related variability. The effect of this procedure on classification performance is varying: DNNs were mostly unaffected by the added noise with mean AUC values of 0.990 (0.989–0.991) and mean PCC values of 0.953 (0.951–0.955) (Fig 4A). SVM and the tree-based RF methodologies are shown to be slightly to moderately impaired by expression data manipulation with average values for AUC decreased to 0.946 (0.944–0.948) and 0.888 (0.883–0.892), respectively, while mean PCC results drop to 0.884 (0.880–0.887) and 0.808 (0.803–0.813) (Fig 4B and 4C). The impact of added noise for DT analysis is much more pronounced: With average AUC and PCC results leveling off at 0.634 (0.627–0.641) and 0.642 (0.636–0.648), DT already displays a poor diagnostic value as a response to the comparatively slight data modification (Fig 4D). Therefore, we did not include other DT results in the following description (Figs 5D and 6D).

(II) Cumulatively to signal modification with noise, the second step of test data degradation introduces random zero-replacement of expression values for 75% of all available genes. For DNNs, the calculated mean AUC and accuracy is 0.964 (0.961–0.967) and 0.906 (0.901–0.910), respectively (Fig 5A). Albeit data manipulation has a small negative effect on the classifier, it has to be noted that just a few results provided by the DNNs differ from the true class labels, considering the vastly decreased number of gene expression data available. Trends shown in the results of RF are comparable to level 1 data degradation: Mean AUC and PCC results are merely unchanged (0.886 (0.881–0.890) and 0.810 (0.805–0.815), respectively) and can be explained by the high number of combined decision trees underlying this classifier (Fig 5C). SVM method exhibits a weakness in response to the limited availability of genes in the testing dataset. Average AUC and PCC values drop to 0.776 (0.764–0.789) and respectively 0.717 (0.706–0.728), implying a marginal diagnostic value for SVM under these circumstances (Fig 5B). Taken together, DNNs possess a robust capacity to diagnose sepsis reliably, even with combined microarray signal interference and limited data availability.

(III) The third step of modification adds randomized substitution of noise-interfered and zero-replaced gene expression data with artificial, yet plausible, expression values for 50% of all available genes. This last level of data degradation mimics the unstructured impact of secondary factors influencing gene expression. As a factual combination of the former two levels with additional artificial data, this randomized modification and replacement of test data could lead machine learning classifiers to misinterpret the remaining true expression values. With this level of severe data modification applied, SVM and RF methods further drop and thus now deliver poor and accordingly marginal performance (Mean AUC: 0.615 (0.604–0.626) and 0.773 (0.764–0.781), respectively; mean PCC: 0.600 (0.592–0.609) and 0.709 (0.701–0.716), accordingly) (Fig 6B and 6C). The performance of DNNs is also strongly affected. However, the demonstrated classification performance is still to be rated best when comparing to the results of the remaining machine-learning-based methods (Mean AUC: 0.818 (0.809–0.826), mean PCC: 0.756 (0.749–0.763)) (Fig 6A), as indicated by statistical comparison (p-value (AUC of SVM vs. DNN) = 1.5×10–71, p-value (AUC of RF vs. DNN) = 2.4×10–12) (Tables 1 and 2).

### Removal of differentially expressed genes does not impact machine learning

In a third experiment, we examined the effect of gene identity on diagnostic performance. Therefore, we excluded 2,361 genes from the meta-expression set, which were identified by differential expression analysis. The residual 3,571 genes (approximately 60%) are processed with all investigated machine-learning-based methods independently in analogy to the second experiment. Classification is again repeated for the native testing data and all levels of data degeneration for 100 iterations while performance is reported. In a detailed comparison of experiments 2 and 3 (Tables 1 and 2), we demonstrate DNN results to remain on a similar performance level. All other machine-learning-based methods are shown to be at least slightly affected by the smaller number of available genes for classification. Except for DNNs, the general diagnostic performance drops as indicated by lower average values of AUC and PCC.

## Discussion

Analysis of differential gene expression is a helpful utility when comparing two well-defined conditions under experimentally controlled circumstances, e.g., identifying disease-related, informative biomarker target genes. In our study, the well-established differential expression analysis method proved to provide valuable results for comparison, especially when combined with hierarchical clustering of experimental samples. Two of the three subgroups identified by the clustering algorithm’s subsequent application discriminated sepsis patients and non-septic samples. In contrast, the existence of a third, unordered cluster containing individuals of both the sepsis class and members of the residual entity indicates limits of differential analysis when applying to a versatile collection of samples. Because of the constraint of focusing on genes that show clear and robust differential expression between both conditions, more subtle but still informative adjustments in gene expression occurring in the entirety or even a subset of samples are disregarded. Thus, the differential expression analysis approach is confined to the most prominent and universal changes by just taking parts of the available information into account. Further downsides of this methodology are the required data preparation steps to adjust for batch effects and the necessity to build a separate regression model for classification based on the identified informative target genes.

The fact that all machine-learning-based methodologies can achieve high-ranking results without the necessity of preceding removal of batch effects [45] or implicit feature selection steps [46] renders them an appealing new option for adaption to high-dimensional data structures. We show a comparably high performance in detecting sepsis patients by combining microarray gene expression data with deep-learning artificial neural networks. The presented results excel both the approach of Jin Kam and Young Kim [47] using a limited set of serial multi-parameter intelligent monitoring in intensive care (MIMIC) II data with DNN methodology (accuracy: 0.915) and the study by Lukaszewski et al. [48]. Their procedure of the deep-learning-based gene expression analysis using RT-PCR data was reported at 0.831, while clinical data alone obtained a lower value of 0.694. The combination of both datasets even decreased predictive accuracy to 0.797. Furthermore, we could partially surpass the results achieved by Dwivedi [49] for the classification of leukemia (accuracy: 0.980), likely because of the considerable differences regarding sample size (46 vs. 3,005) and the number of genes included (7,129 vs. 5,932).

Using DNNs, the here demonstrated classifier offers the capability to reliably distinguish between immunological phenotypes of sepsis and non-sepsis, although a variety of genes is known to be commonly dysregulated in response to, e.g., trauma conditions [50]. Due to the striking similarities to the sepsis signature that can be observed at the molecular level for parts of the non-sepsis entity, we propose successful differentiation between sepsis and phenotypes of SIRS and trauma to be the ultimate stress test for any gene expression-based diagnostic method. For successful classification, detection of slight differences in early transcriptional response to proinflammatory signals is required. By neural network adaptation, we can identify and process idiosyncrasies of sepsis and the variety of non-sepsis response signals to create a robust diagnosis methodology. For that reason, we propose that microarray-based DNNs exceed most other available diagnostic solutions regarding sepsis, SIRS, and trauma differentiation on the molecular level.

Instead of finding a general solution for classification, machine-learning-based classifiers can overfit sample data during model training. Application of an overfit model to unknown or imperfect data results in low classification performance compared to training results. Thus, we performed resilience testing for all machine-learning-based classifiers by simulating biological variation and technical difficulties affecting measurements, e.g., missing data or differences between microarray platforms. Since data removal has no substantial effect on the results of DNNs, we suggest that by the utility of two dropout layers during the neural network training phase, identification of a multitude of redundant strategies for sepsis classification has been enforced for this classifier. Our findings indicate that a manifold of complex relationships between various genes should enable us to diagnose sepsis safely instead of drawing upon single genes. L2 kernel regularization during neural network training causes high weight values to be penalized by adding their squared values as error to the loss function. Because weights have linear effects on prediction results, a focus on particular genes does not occur in the resulting classifier. Therefore, the here shown implementation for sepsis diagnosis with DNNs differs from well-established approaches based on a defined set of biomarker genes [48, 51]. In this context, regularized neural network information processing follows the ideas of applying gene networks to microarray data classification [52], although no precedent knowledge of the underlying relationships is required.

When comparing the runtime for differential gene expression analysis, which can be calculated without delay using modern hardware, creating a neural network classifier is far more time-consuming and computationally extensive. Thus, the iterative training of DNNs often requires special hardware features found in modern GPUs or more advanced solutions to speed up computation. For the actual classification of new samples, pre-trained networks are accessed. Therefore, even on standard performance hardware, the runtime leading to final diagnostic results ranges merely in the magnitude of seconds.

Limits of the underlying meta-expression set primarily originate from the diverging numbers of samples between the data series included and the amount of variance introduced. Compensating this data in-heterogeneity by gathering additional microarray samples or applying novel prospective data augmentation strategies could most probably lead to close-to-perfect classification results. Furthermore, since the microarray platforms selected for this publication substantially differ between the number and the contained genes, we are forced to limit neural network input genes to the least common denominator between all platforms. Based on the obtained results, we can predict, given a hypothetical homogeneous expression set with a reasonable number of samples and an unrestricted number of gene expression parameters (~20,000), DNNs will most likely achieve perfect classification performance to distinguish septic patients from non-septic samples.

The standard procedure of blood sample collection and laboratory analysis must be extended by microarray gene expression measurement and additional computation-based analysis to take the suggested method’s diagnostical advantage. Apart from microarray kits and equipment requirements on the lab-side, a regular server system for hosting neural network classification software would be necessary to facilitate in silico diagnoses. The actual diagnosis of a potentially new and unknown expression set could be executed in a four-step process by (1) limiting the available expression values of the new data to the defined standard set of 5,932 genes. Further, to ensure predictability, newly collected expression values had (2) to be centered gene-wise based on the mean values of the full meta-expression set, and (3) scaled by its standard deviation. After preparation, the expression values are required to be (4) processed inside a pre-trained neural network environment, resulting in an instant prediction of sepsis occurrence, even during the temporary availability of fractions of the complete gene expression data. The here proposed methodology could therefore offer precise predictions of sepsis in real-time.

With “SeptiCyte LAB”, the first host response gene expression assay for diagnosis of sepsis, has recently passed the U.S. Food and Drug Administration (FDA) for validation studies with AUC results reported ranging between 0.82 to 0.89 depending on the degree of confidence in the clinical diagnosis [53]. Besides SeptiCyte, two other solutions for the gene-expression-based sepsis diagnosis have been benchmarked: The Sepsis MetaScore and the FAIM3-to-PLAC8 ratio were reported to discriminate sepsis patients from patients with non-infectious inflammation at AUC scores of 0.82 (0.73–0.89) and 0.78 (0.49–0.96), respectively. For diagnosing sepsis from a combined collection of septic patients and healthy controls, AUC values were ranked at 0.97 (0.85–1.0) for the Sepsis MetaScore and 0.94 (0.65–1.0) in the particular case of the FAIM3-to-PLAC8 ratio [54]. Since the alternative approach presented here shows very high performance compared to established solutions for molecular sepsis diagnosis, we propose that combining standardized gene expression assays with custom-built DNN solutions could revolutionize early sepsis recognition. Furthermore, a reliable and effective diagnostic toolset is essential for more sophisticated developments in personalized intensive medicine.

## Conclusions

We trained deep-learning artificial neural networks and other machine-learning-based classifiers with microarray gene expression data with the goal of sepsis classification for this publication. Referring to accuracy, the proposed approach using DNNs exceeds solutions interpreting EHR data based on a comparable number of samples [47] and RT-PCR measurements [52], respectively. Published solutions for gene-expression-based sepsis diagnosis are partially surpassed based on published AUC scores [53, 54]. The results suggest that the methodology, even if a fraction of the original gene expression training signal is available for classification, can offer reliable discrimination of immunological highly similar conditions like sepsis, SIRS, and trauma. In summary, the proposed solution substantially augments current diagnostic capability in intensive care medicine.

## Supporting information

S1 File Overview of collected samples. (DOCX)

S2 File Overview of software components. (DOCX)
