# Supplementary material for: Comparison of machine-learning methodologies for accurate diagnosis of sepsis using microarray gene expression data
Source: PLoS One. 2021 May 17;16(5):e0251800. doi: 10.1371/journal.pone.0251800 (PMC8128240; doi:10.1371/journal.pone.0251800)
Supplement: S1 File — (DOCX) [file pone.0251800.s001.docx]

S1 File.

Comparison of machine-learning methodologies for accurate diagnosis of sepsis using microarray gene expression data

Dominik Schaack^1*^, Markus A. Weigand^1^, Florian Uhle^1^

^1^ Department of Anesthesiology, Heidelberg University Hospital, Heidelberg, Germany

* Corresponding author

E-mail: dominik.schaack@med.uni-heidelberg.de (DS)

**S1 File. Overview of collected samples.**

| **ID** | **First author** | **Year** | **Type** | **Vendor** | **Platform** | **Sepsis** | **Trauma** | **SIRS** | **Control** |
| --- | --- | --- | --- | --- | --- | --- | --- | --- | --- |
| GSE54514 | Parnell [8] | 2013 | Case-control study | Illumina | HumanHT-12 V3.0 expression beadchip | 35 | 0 | 0 | 18 |
| GSE74224 | McHugh [9] | 2015 | Observational study | Affymetrix | HuEx-1_0-st | 74 | 0 | 31 | 0 |
| GSE65682 | Scicluna [10] | 2015 | Case-control study | Affymetrix | HG-U219 | 108 | 0 | 0 | 42 |
| GSE66890 | Kangelaris [11] | 2015 | Observational study | Affymetrix | HuGene-1_0-st | 57 | 0 | 0 | 0 |
| GSE57065 | Cazalis [12] | 2014 | Case-control study | Affymetrix | HG-U133_Plus_2 | 28 | 0 | 0 | 25 |
| GSE33341 | Ahn [13] | 2013 | Case-control study | Affymetrix | HG-U133A_2 | 51 | 0 | 0 | 43 |
| GSE32707 | Dolinay [14] | 2012 | Case-control study | Illumina | HumanHT-12 V4.0 expression beadchip | 48 | 0 | 21 | 0 |
| GSE28750 | Sutherland [15] | 2011 | Case-control study | Affymetrix | HG-U133_Plus_2 | 10 | 0 | 0 | 20 |
| GSE13015 | Pankla [16] | 2009 | Case-control study | Illumina | HumanHT-12 V3.0 expression beadchip | 13 | 0 | 0 | 5 |
| GSE10474 | Howrylak [17] | 2009 | Observational study | Affymetrix | HG-U133A_2 | 34 | 0 | 0 | 0 |
| E-MTAB-4421 | Davenport [18] | 2016 | Cohort study | Illumina | HumanHT-12 V4.0 expression beadchip | 265 | 0 | 0 | 0 |
| E-MTAB-4451 | Davenport [18] | 2016 | Cohort study | Illumina | HumanHT-12 V4.0 expression beadchip | 106 | 0 | 0 | 0 |
| E-MTAB-5273 | Burnham [19] | 2017 | Cohort study | Illumina | HumanHT-12 V4.0 expression beadchip | 80 | 0 | 0 | 10 |
| E-MTAB-5274 | Burnham [19] | 2017 | Cohort study | Illumina | HumanHT-12 V4.0 expression beadchip | 53 | 0 | 0 | 0 |
| GSE95233 | Venet [20] | 2017 | Cohort study | Affymetrix | HG-U133_Plus_2 | 51 | 0 | 0 | 22 |
| GSE26440 | Wong [21] | 2009 | Case-control study | Affymetrix | HG-U133_Plus_2 | 98 | 0 | 0 | 32 |
| GSE26378 | Wynn [22] | 2010 | Case-control study | Affymetrix | HG-U133_Plus_2 | 75 | 0 | 0 | 21 |
| GSE25504 | Smith [23] | 2014 | Case-control study | Affymetrix; Illumina; Codelink | HG-U133_Plus_2, HG-U219; HumanHT-12 V3.0 expression beadchip; 55K Human Array | 63 | 0 | 0 | 71 |
| GSE13904 | Wong [24] | 2009 | Observational study | Affymetrix | HG-U133_Plus_2 | 16 | 0 | 15 | 3 |
| GSE9692 | Cvijanovich [25] | 2008 | Cohort study | Affymetrix | HG-U133_Plus_2 | 30 | 0 | 0 | 0 |
| GSE4607 | Wong [26] | 2007 | Cohort study | Affymetrix | HG-U133_Plus_2 | 42 | 0 | 19 | 15 |
| E-MTAB-4785 | Cernada [27] | 2014 | Case-control study | Affymetrix | HuGene-1_0-st | 17 | 0 | 0 | 19 |
| GSE36809 | Xiao [29] | 2011 | Cohort study | Affymetrix | HG-U133_Plus_2 | 0 | 185 | 0 | 35 |
| GSE37069 | Peterson [30] | 2014 | Cohort study | Affymetrix | HG-U133_Plus_2 | 0 | 248 | 0 | 0 |
| GSE19743 | Zhou [31] | 2010 | Cohort study | Affymetrix | HG-U133_Plus_2 | 0 | 57 | 0 | 63 |
| GSE77791 | Plassais [32] | 2017 | Randomized controlled trial | Affymetrix | HG-U133_Plus_2 | 0 | 30 | 0 | 13 |
|  |  |  |  |  |  |  |  |  |  |
|  |  |  |  |  | **Total:** | **1354** | **520** | **86** | **457** |
