# Supplementary material for: Comparison of machine-learning methodologies for accurate diagnosis of sepsis using microarray gene expression data
Source: PLoS One. 2021 May 17;16(5):e0251800. doi: 10.1371/journal.pone.0251800 (PMC8128240; doi:10.1371/journal.pone.0251800)
Supplement: S2 File — (DOCX) [file pone.0251800.s002.docx]

S2 File.

Comparison of machine-learning methodologies for accurate diagnosis of sepsis using microarray gene expression data

Dominik Schaack^1*^, Markus A. Weigand^1^, Florian Uhle^1^

^1^ Department of Anesthesiology, Heidelberg University Hospital, Heidelberg, Germany

* Corresponding author

E-mail: dominik.schaack@med.uni-heidelberg.de (DS)

**S2 File. Overview of software components.**

| **Name** | **Version** |
| --- | --- |
| R | 3.6.2 |
| Bioconductor | 3.10 |
| sva | 3.34.0 |
| limma | 3.42.2 |
| tree | 1.0-39 |
| e1071 | 1.7-3 |
| randomForest | 4.6-14 |
| TensorFlow | 2.0.0 |
| keras | 2.2.5.0 |
| PresenceAbsence | 1.1.9 |
